# Metadata Framework to Support Deployment of Digital Health Technologies in Clinical Trials in Parkinson’s Disease

**DOI:** 10.3390/s22062136

**Published:** 2022-03-09

**Authors:** Derek L. Hill, Diane Stephenson, Jordan Brayanov, Kasper Claes, Reham Badawy, Sakshi Sardar, Katherine Fisher, Susan J. Lee, Anthony Bannon, George Roussos, Tairmae Kangarloo, Viktorija Terebaite, Martijn L. T. M. Müller, Roopal Bhatnagar, Jamie L. Adams, E. Ray Dorsey, Josh Cosman

**Affiliations:** 1 Panoramic Digital Health, 38000 Grenoble, France; 2 Centre for Medical Imaging, University College London (UCL), London WC1E 6BT, UK; 3 Critical Path Institute, Tucson, AZ 85718, USA; dstephenson@c-path.org (D.S.); ssardar@c-path.org (S.S.); mmuller@c-path.org (M.L.T.M.M.); rbhatnagar@c-path.org (R.B.); 4 Takeda Development Center Americas, Inc., Deerfield, IL 60015, USA; jordan.brayanov@takeda.com (J.B.); tairmae.kangarloo@takeda.com (T.K.); 5 UCB Pharma, 1070 Brussels, Belgium; Kasper.Claes@ucb.com; 6 School of Computer Science, University of Birmingham, Birmingham B15 2TT, UK; rehambadawy@hotmail.com; 7 Biogen, Cambridge, MA 02142, USA; katherine.fisher@biogen.com; 8 Merck, Kenilworth, NJ 07033, USA; susi_lee@merck.com; 9 AbbVie, North Chicago, IL 60064, USA; anthony.bannon@abbvie.com (A.B.); josh.cosman@abbvie.com (J.C.); 10 Birkbeck College, University of London, London WC1E 7HX, UK; g.roussos@bbk.ac.uk; 11 H.Lundbeck A/S, 2500 Valby, Denmark; VITR@lundbeck.com; 12 Department of Neurology, University of Rochester, Rochester, NY 14642, USA; Jamie.Adams@chet.rochester.edu (J.L.A.); Ray.Dorsey@chet.rochester.edu (E.R.D.)

**Keywords:** digital health technology, Parkinson’s disease, metadata standards

## Abstract

Sensor data from digital health technologies (DHTs) used in clinical trials provides a valuable source of information, because of the possibility to combine datasets from different studies, to combine it with other data types, and to reuse it multiple times for various purposes. To date, there exist no standards for capturing or storing DHT biosensor data applicable across modalities and disease areas, and which can also capture the clinical trial and environment-specific aspects, so-called metadata. In this perspectives paper, we propose a metadata framework that divides the DHT metadata into metadata that is independent of the therapeutic area or clinical trial design (concept of interest and context of use), and metadata that is dependent on these factors. We demonstrate how this framework can be applied to data collected with different types of DHTs deployed in the WATCH-PD clinical study of Parkinson’s disease. This framework provides a means to pre-specify and therefore standardize aspects of the use of DHTs, promoting comparability of DHTs across future studies.

## 1. Introduction

Digital health technologies (DHTs) are attracting considerable interest in clinical trials of new treatments for Parkinson’s disease (PD). These technologies have many potential advantages that complement traditional clinical assessments, including high-frequency data collection, improved objectivity, the ability to capture occasional events such as freezing of gait, and more naturalistic data collected in a home setting [1]. There remain, however, many challenges before these technologies can significantly impact clinical trial design and practice. One of these challenges is the comparability of data, e.g., to make it possible to compare results from different studies of PD patients for meta-analysis and as evidence for the efficacy of a digital health technology. This requires improved standardization of data collection and analysis, and also the harmonization of the requirements for unique data types from a potentially wide range of digital health technologies, each of which may have been deployed and setup in different ways. Such standardization efforts have previously been used in other drug development tool technologies such as neuroimaging [2] and gene expression microarrays [3]. Data standards that are limited to a single modality exist, such as for ECG: SCP-ECP, DICOM-ECG and HL7‘s aECG [4]. Similarly limited to a single modality are electrophysiology data standards such as MEF [5,6], Brainformat and Neurodata Without Borders [7]. There are metadata standards that have been proposed for the output of digital technologies used for patient management, and integration with the Electronic Health Record (including IEEE P1752.1 [8] and OHDSI [9].

However, data standardization and harmonization (i.e., the identification of common requirements for data collection and analysis) for DHTs used in clinical research is in its infancy. While true standardization will take time, progress in this area is likely to accelerate the regulatory acceptability of measures derived from DHTs.

Advancing of the regulatory maturity of measurements derived from DHTs is a particular focus of the Critical Path for Parkinson’s Digital Drug Development Tools (3DT) consortium, and the absence of a suitable metadata framework for standardizing the way measurements are made using DHTs in clinical trials was identified as one of the key barriers to advancing their widespread use in clinical studies intended for submission to health regulators. Metadata is the data needed to interpret the data (such as motion sensor data) collected by a DHT. A crucial step towards standardization and harmonization of DHTs for use in endpoint development is defining the metadata needed to describe how the data used to produce the study endpoint has been generated. For example, the DHTs we are considering in this manuscript collect data from one or more individual data collection devices (e.g., wrist-worn devices or smartphones containing inertial measurement units (IMUs), microphones, PPG or ECG sensors). Each of these types of sensors can be implemented in different hardware, can be incorporated into devices with different form factors, can be worn in different body locations, and can be configured and pre-processed by firmware and software in numerous ways before the sensor data is output from the device.

Comprehensive metadata is needed to describe how data from an individual DHT is collected in order to properly interpret the DHT output and compare with similar data from a different DHT or the same DHT configured in a different way. Further metadata is needed to describe how the DHT is deployed in a particular clinical application such as a clinical trial of a new treatment for early PD. This additional metadata needs to detail how the clinical trial was performed, such as the clinical population being studied, whether the DHT collected data “passively” or during performance of standardized tasks (“actively”), and any application-specific data analysis undertaken. Previously, a metadata concept for advancing the use of digital health technologies in Parkinson’s disease has been proposed in Badawy et al., 2019 [10]. The framework we present here builds on that work in the following ways. Firstly, it divides the metadata into the two categories introduced above: *application independent*, and *application dependent*. Secondly, we propose that this metadata framework can go beyond describing the DHT data, but also has the potential to be used to pre-specify how data is collected in future clinical trials in order to help standardize DHT use across studies. In the regulatory language of drug development tools, the application-specific metadata is associated with the *concept of interest* and *context of use* [11,12].

Once a metadata framework has been defined and implemented, it provides a way to fully describe how DHT outputs have been obtained, which will facilitate data sharing and collaboration as well as reproduction of the measure in future studies wishing to use a given endpoint generated by a DHT. Using a metadata framework to pre-specify how data is collected could also enable new ways of capturing and controlling the sources of variability that are described in a related publication from this consortium [13]. Taken together, such a metadata framework could be used to advance the regulatory maturity of endpoints derived from DHTs and pave the way for more widespread use in therapeutic development.

In this paper, we describe how metadata has helped to advance regulatory maturity of other drug development tools. We then propose a metadata framework, which was developed by members of the Critical Path for Parkinson’s consortium (CPP). Formulation of this framework took into account approaches used for other drug development tools along with our own independent efforts which were informed by the specific feedback we obtained from health regulators in the US and Europe [14]. The proposed framework is then applied to an example study in Parkinson’s disease, namely the measurement of tremor using both research-grade and consumer-grade wearable sensors in the WATCH-PD study NCT03681015 [15]. Although presented in the context of Parkinson’s disease, the new framework is intended to be applicable to DHTs used in observational studies and other therapeutic area.

## 2. Metadata and Standardization of Drug Development Tools: Learning from Neuroimaging

A successful example of standardization and harmonization of digital data with a similar degree of complexity to that of DHTs is neuroimaging in Alzheimer’s disease clinical trials. An aim of the Alzheimer’s Disease Neuroimaging Initiative (ADNI) project was to make neuroimaging measurements more acceptable to regulators for evaluating novel treatments for Alzheimer’s disease. A key goal of ADNI was therefore to use MRI scanners and PET scanners at multiple hospitals with different hardware and software configurations, to make comparable measurements of disease-related change in the brains of Alzheimer’s disease patients. The starting point was an experimental process to standardize the way the data was collected from a pre-determined range of MRI and PET scanners manufactured by different companies (Siemens, GE, Philips), to obtain comparable results for some defined metrics of brain volume change (whole-brain volume, ventricle volume, hippocampal volume) and brain function [2,16]. Amyloid PET imaging using Fluorinated tracers was later added to the ADNI project [17].

Key aspects of the standardized approach to neuroimaging data in AD are (1) a description of all the MRI and PET scanning parameters, augmented by a detailed set of instructions on how patients should be prepared and positioned for scanning, (2) how data quality assurance and handling should be performed, and (3) defining the clinically meaningful derived measures that were suitable as study endpoints (e.g., hippocampal volume and whole-brain atrophy from structural MRI, Cortical Average Standardized Uptake Value Ratio from amyloid PET). This example from a different field demonstrates that obtaining comparable data from heterogeneous data collection devices involved both defining the *application-independent* metadata that described how data was collected from those devices (in this case MRI and PET scanners), and *application-dependent* metadata describing how these are used for deriving a measure relevant to the desired clinical trial application. The increasing regulatory maturity of imaging endpoints in Alzheimer’s disease clinical trials, which the ADNI project helped standardize, is illustrated by the recent accelerated approval of aducanumab by the FDA. Accelerated approval is an FDA mechanism to approve a novel drug in an area of important medical need, based on the drug’s effect on a “surrogate endpoint” that is reasonably likely to predict a clinical benefit to patients, rather than a more traditional clinical endpoint. In the case of aducanumab, the surrogate endpoint was amyloid positron emission tomography standardized uptake value ratio (PET SUVR), which the FDA concluded showed that the drug provided a dose-and time-dependent reduction in amyloid beta plaque in patients with Alzheimer’s disease [18].

## 3. Proposed Metadata Framework for DHTs

**Figure 1:**
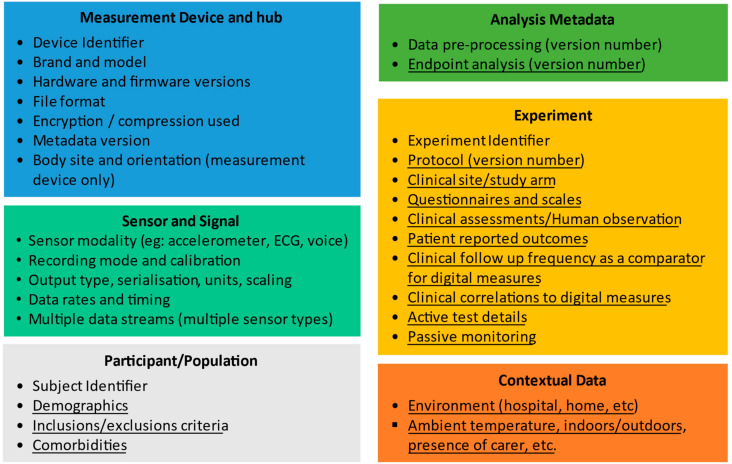
A summary of the metadata elements needed to describe the collection of data from digital health technologies in a clinical trial setting. Underlined elements are application dependent and non-underlined items are application independent. The Patient ID and Experiment ID in the application-dependent metadata link to the application-dependent metadata. The “device” elements are required for the measurement device (e.g., a wearable) but also hub (might be a smartphone + app) that works with the wearable.

The essential types of metadata that should be collected during a clinical trial to describe the process in appropriate detail is provided in Figure 1. These metadata types can be divided into six distinct categories:

- Measurement Device and Hub metadata,
- Sensor and signal metadata,
- Participant/Population metadata,
- Analysis metadata,
- Experimental metadata and
- Contextual metadata

Some of this metadata is quite generic and can be used to describe data collected for many clinical applications. For example, a wearable actigraphy device could be used to measure many different parameters in different patient populations, including total activity, gait speed, turning gait, falls, sleep, and tremor. For all these applications, there is therefore a core set of *application-independent metadata* that describes the sensor(s) and signals, and the data collection hardware and software.

Other metadata is specific to a particular clinical application and is required, e.g., to compare or combine measurements from DHTs obtained in different clinical trials for the same therapeutic area, and we refer to this as *application-dependent metadata*. In Figure 1, we distinguish between these two types by using underlining to indicate the application-dependent metadata, and with application-independent metadata not underlined.

### 3.1. Pre-Specification of Metadata

To support standardization and quality control of DHT data in clinical trials, we propose that the values of the metadata required for a particular study should be specified a priori. In this way, the metadata framework does not just retrospectively describe data that has already been collected but can be used to define how it should be collected and enable quality assurance of that data collection. Furthermore, by using the same pre-specified values for different studies, such pre-specification will support standardization of measurement across trials, and re-use of analytically validated DHT methodology. For example, the application-independent metadata might pre-specify the desired data acquisition rate, the location and orientation of a wrist worn accelerometer, and the required hardware and firmware version. The application-dependent metadata could specify any Patient-Reported Outcome (PRO) or clinical assessment completed simultaneously with the data collection, and the time of day and expected duration of any specific “active” tasks performed by the subjects. These pre-specified values could then be used to setup the DHTs being deployed in this study and could also be used for an automatic quality check that the collected data contains the pre-specified characteristics.

In the same way, we carefully specify the exact sequence of events, tests, procedures, and measurements during the execution of a clinical trial, we should also specify the *minimum requirements* for any data coming from DHTs, including the metadata, such that we can ensure successful completion of this study. We propose that this detail should be provided in a dedicated DHT Charter that might be annexed to the protocol, or treated like the Independent Review Charters used to standardize imaging endpoints in clinical trials [19].

### 3.2. Application-Independent Metadata

In the DHT metadata elements listed in Figure 1, a subset of metadata is identified as independent of the clinical application (these are the elements that are not underlined). This application-independent metadata is therefore generic, which is appropriate, as many digital technologies are applied to multiple clinical applications and clinical trial concepts of interest. For example, a smartwatch could be used to assess sleep, gait, or tremor. In all cases, to properly characterize the way data has been collected, it is important to have the following application-independent metadata elements:

- **Measurement device and hub**: Comprises metadata that uniquely identifies the:
  ○ **Measurement device** used for data collection, including its brand, model, serial number (medical device UDI where available), hardware and firmware version. This metadata needs to allow the device location on the body and orientation to be recorded. Furthermore, by tracking individual device ID, any change in performance over time or repairs can be associated with the data.
  ○ **Hub**: Since many wearable measurement devices (e.g., smartwatch) work in combination with a separate device (e.g., smartphone or more generically, a data hub) in order to interconnect with a remote database and potentially also to perform other functions such as pre-processing and authentication, the application-independent metadata also includes metadata to uniquely describe the hardware/software of the hub which the measurement device connects.
  ○ The **file format** and technical aspects of the data storage and transfer (compression, encryption).
  ○ **Metadata version.** The metadata framework needs to be able to be refined so it is important that there is a metadata version associated with the device collecting data.
- **Sensor and Signals**: is the description of the types of data collected including the modality (e.g., accelerometer, EEG/electroencephalogram, ECG/electrocardiogram, PPG/photoplethysmogram), the recording mode and any calibration of the sensor performed prior to deployment in each study, data rates and timing. A single DHT may generate multiple signals with distinct metadata, for example, a DHT might include an accelerometer, gyroscope, magnetometer and PPG sensor, each operating at different acquisition frequency and with different timing information. The metadata framework supports this through a single device supporting multiple sets of sensor metadata. The signals can cover traditional wearable sensor signals, but may also be used for environmental context signals, such as the ambient temperature where subject is located, whether the subject is indoors or outdoors (this could be a binary signal), which room in their home they are located in (the signal would be a number specifying the room identity) and whether they are alone in that room or accompanied.
- **Participant/Population**: We propose that the application-independent metadata has a single element of participant/population metadata, namely a unique identifier that can be linked to this subject data in the application-dependent metadata.
- **Analysis metadata**: The application-independent metadata needs to describe any generic analysis performed in the device itself (e.g., the device might output step count or heart rate variability), which we refer to as “pre-processing” to distinguish from endpoint-specific analysis that is application dependent.
- **Experiment metadata:** We propose that the only application-independent metadata element for the experimental metadata is an experiment identifier. The details of the experiment being performed are application dependent.

A practical benefit of this approach is that the application-independent metadata is compact and generic, while at the same time, being closely associated with the application-dependent metadata described in the next section.

### 3.3. Application-Dependent Metadata

The application-independent metadata alone is not sufficient to be able to reproduce the experimental context in which a given measurement was completed. The required metadata needs to take into account the underlying clinical sign or symptom, or biological process (i.e., the ‘concept of interest’) that is being measured using the DHT. We call this additional metadata the “application-dependent metadata.”

#### 3.3.1. Implementation of Application-Dependent Metadata

The application-dependent metadata format in the framework shown in Figure 1 is necessarily flexible to handle the variety of types of sensors, therapeutic areas, and clinical trial designs that may apply. Pragmatically, our framework does not require that the application-dependent metadata is duplicated in a dedicated DHT dataset. It may be more appropriate to link to existing sources of the required information, for example in the clinical trial database, clinical trial protocol, or data analysis plan, or a dedicated DHT charter as proposed earlier. In the latter case, it is important that a means is implemented to extract the required linked metadata when necessary to enable the study data to be shared or aggregated. The aspects of application-dependent metadata in Figure 1 are described as follows:

- **Subject metadata**: The application-independent metadata only includes a subject unique identifier (UID). The application-dependent metadata includes the relevant demographics and associated health information (e.g., medical history) relevant to the clinical study concerned, the inclusion and exclusion criteria and any comorbidities relevant to this study.
- **Analysis metadata**: The data analysis is in many cases very specific to the clinical trial design. We refer to this application-dependent analysis as “endpoint analysis” to distinguish from the generic pre-processing described in the application-independent metadata. All relevant software versions and selectable parameters must be clearly defined.
- **Experiment metadata**: This describes the clinical trial cohort in which a given subject is enrolled, the clinical site, any clinical trial questionnaire or human (e.g., physician) observation metadata, and a reference to the applicable protocol and its version number. In particular, this metadata needs to include details of any active tests and passive monitoring involved, and the details of the active test.
- **Contextual data**: A description of the environment of data collection (e.g., clinic, home) and properties of the environment (such as ambient temperature, noise level and light level) if available should be included in the metadata.

#### 3.3.2. Challenges of Application-Dependent Metadata

The application-dependent metadata aspect of the framework is flexible to support use in multiple clinical trial designs in PD and beyond. Some key challenges in implementing application-dependent metadata, and the way in which this framework addresses these, are listed below.

1. **Variability in metadata requirements across clinical applications and sensor modalities**: For example, we may be interested in monitoring gait in patients with Parkinson’s disease. In one specific clinical trial, we may want to evaluate a patient’s gait using a wrist-worn wearable device in a clinical setting during the performance of a 6-min walk test. In that instance, it may be necessary to record, as metadata, the actual length of the lab or walkway that the patient is using for the test and whether the test was performed with or without caregiver support. This application-dependent metadata would not be required if one would like to evaluate the same patient’s gait at home. Similarly, in some clinical trials, data might be acquired continuously (passively), and for other applications, data would be collected when the subject is prompted to perform a task or complete a PRO (active). For active tasks, the application-dependent metadata would need to include a description of the prompt or simultaneous PRO to fully describe the data collection. The metadata framework proposed here incorporates the necessary detail in the experiment metadata portion of the application-dependent metadata.
2. **Variability in metadata requirements across different stages of PD:** Severity of disease would also have a significant impact on the metadata that should be recorded within a particular trial. If studying individuals with probable PD in the pre-manifest stage of the disease, there may be minimal motor symptoms, and as such subjects may often engage in vigorous activities such as running that would be captured by a continuously recording activity monitor. This would not be the case for patients with advanced disease, who may struggle to safely and independently navigate their own homes. Thus, if we were to devise a measure of “average daily activity” it would greatly vary across these two populations. In the case of pre-manifest PD, a study might measure the amount of time of moderate-to-vigorous activity per week and in the case of advanced PD, a study might seek to measure any and all activity. In the former group, we would need to capture extraneous factors that may have impacted a patient’s ability to perform vigorous activity: if we are monitoring a golfer who usually plays 2–3 times/week, a month-long weather pattern may substantially alter their activity levels. In the latter group, these factors may not be as relevant. The flexible design of the application-dependent metadata format in the proposed framework allows this variability to be described in the experiment metadata and Analysis metadata.
3. **Data pre-processing**: Another challenge we face is that DHTs do not always provide ready access to the **raw data**, as we are used to collecting from research-grade clinical equipment. Additionally, even if there is access to some version of the raw data, these data often vary greatly across devices, based on the manufacturer or even the version of a specific device. Smartwatch actigraphy devices that generally report step count over a defined epoch often claim to also output raw data that we hope to use for clinical research. Indeed, the term “raw data” is seldom the output of the analogue to digital converters (ADCs) in the sensor, but normally has filters or data compression applied and is often the output of a software interface (API) provided by the manufacturer. Data streams with such differences may not be used interchangeably. The application-independent metadata in our proposed framework addresses this challenge by including both sensor metadata fields that records software and hardware versions and the Device ID. The Device section of the application-independent metadata in the framework therefore uniquely identifies the type of pre-processed data available, even when the details of the pre-processing are not provided by the manufacturer. Given precise specification of this device metadata, lab-based experiments can be used to test whether the data output from different hardware/software versions of the same device are sufficiently similar to be combined in a particular context.
4. **Data analysis**: In addition to pre-processing on the wearable hardware itself, DHTs involve analysis to calculate measurements of interest from the sensor data. This analysis is normally done after pre-processing, and on one or more separate devices such as a mobile phone app, a home hub, or cloud server. Data analysis software can evolve and be changed, and indeed some of this type of analysis software is “self-learning” and changes how it works in use. The proposed framework addresses this challenge through a detailed description of all pre-processing in the application-independent metadata, including details of hardware and software versions of any smartphone app or hub that is used as an intermediary between a data collection device and the data analysis platform. The application-dependent metadata in our framework then uniquely identifies the data analysis software, including its version number that is applied to this input.
5. **Controlling environmental sources of variability**: The environment of data collection is an important source of variability. For example, a clinical trial subject’s mobility may depend on the ambient temperature as well as their symptoms and treatment, and behaviours and activities are influenced by whether the clinical trial subject is on their own or in the same room as a family member or care partner. The way a subject walks may also depend on whether they are inside or outside their home, or which room in the house they are in. A timed up and go task will be influenced by the height of the chair from which the subject stands up. Increasingly, clinical trials are capturing information about these environmental factors. In our framework, such environmental data is captured as a separate sensor (e.g., temperature) but other environmental context would more appropriately be captured in an experiment metadata portion of the application-dependent metadata, for example whether a particular assessment is being performed in the clinic or at home, and whether it is done as part of a prompted task or passively.

Such challenges and limitations cause difficulties in many clinical studies that deploy digital health technologies, mostly stemming from lack of standardization in the devices and their output. We believe our proposed framework is a practical approach to dealing with these challenges, as it defines both the metadata that any researcher should carefully specify, collect, and record about the clinical study design in addition to the metadata about the sensor dataset provided by the devices.

## 4. Use Case Example—Tremor in Parkinson’s Disease

We illustrate the application of this metadata framework to the measurement of tremor in Parkinson’s disease, using information from the Wearable Assessments in the Clinic and Home in PD (WATCH-PD) study [15]. In particular, we show how the metadata framework supports pre-specification and therefore standardization of data collection, as well as comparisons of data for the same concept of interest collected from different types of sensors.

WATCH-PD is a 12 month multicenter, longitudinal, digital assessment study of PD progression in subjects with early untreated PD (clinicaltrials.gov #NCT03681015). The primary goal is to generate and optimize a set of candidate objective digital measures to complement standard clinical assessments in measuring the progression of PD and the response to treatment. A secondary goal is to understand the relationship between standard clinical assessments, research-grade digital tools used in a clinical setting, and more user-friendly consumer digital platforms to develop a scalable approach for objective, sensitive, and frequent collection of motor and nonmotor data in early PD.

In WATCH-PD, two different DHTs were deployed in the same subjects, and both were capable of measuring tremor–patients wore both the APDM (Ambulatory Parkinson’s Disease Monitoring) Opal sensors and an Apple watch while completing a standardized set of tremor tasks in a clinic setting.

**Figure 2:**
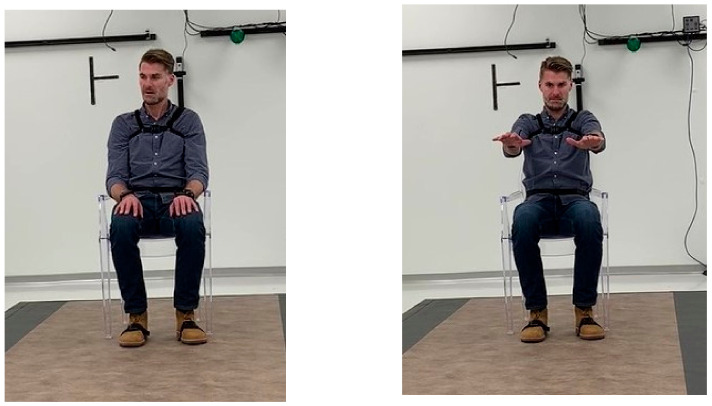
Illustration of WATCH-PD digital sensor instrumentation during defined motor examination test as demonstrated by co-author JC.

Participants were instrumented with both the six-sensor opal system recording continuously and an Apple watch placed on their most affected side. Subjects initiated recordings on the watch using a paired mobile application. Participants were instructed while seated to hold two positions (Figure 2) for 30 s: hands resting comfortably on their lap, and then arms out in front of them with their palms down.

An example metadata framework for tremor measurement based on that in Figure 1 is shown in Table 1 below, with the elements populated based on the WATCH-PD tremor measurement protocol outlined above. As has been previously described, the metadata framework defines the metadata that should be used but does not specify the data format of that metadata.

## 5. Discussion

We have described challenges of using DHTs in clinical trials that could be mitigated with an appropriate metadata framework and have proposed such a framework to help address the challenges and enhance the utility of DHTs in drug development and other clinical and research applications. While the exemplar application given is for Parkinson’s Disease, we propose that this framework is more generally applicable where DHTs are used in observational or therapeutic studies and in patient management. The proposed metadata framework divides metadata into two classes: metadata independent of the clinical trial design (application-independent metadata), and metadata dependent on the clinical trial design (application-dependent metadata). Our framework proposes a method for linking these classes of metadata, and we have provided an example of how the framework can be applied to the measurement of tremor using two different types of sensor platform deployed in the WATCH-PD study.

A particular innovation in this metadata framework is that it is designed to support pre-specification of the minimum required values of the relevant metadata fields, and by comparing pre-specified values with actual values provides a quality assurance framework for data collected using DHTs. The framework proposes that the pre-specified values of both application-independent and application-dependent metadata (where applicable) are documented in a dedicated DHT Charter that describes both how DHT data *should be* collected, and also how it was collected.

A further challenge in DHT metadata is to capture the environmental context in which data was collected. For example, a measurement of gait speed may be influenced by disease progression or effective treatment, but it is also influenced by whether the clinical trial subject is inside or outside, the ambient temperature, the size of the room they are in, etc. This environmental context is a key source of variability in data from DHTs deployed in clinical trials and is an active area of research as well as a topic of attention by regulatory agencies [14,20]. Although environmental context data was not available for the WATCH-PD study, the metadata framework described in this paper supports the inclusion of environmental context metadata when such metadata is available, with this information being included as a further “signal” type in the DHT metadata.

There are several limitations to the approach outlined in this paper. First, the WATCH-PD study is just one exemplar and this study has not yet been completed at the time of submission of this manuscript. The metadata from this one study may not be generalizable to other DHT studies collecting data on similar concepts of interest from different devices. The ability to generalize this framework will need to be tested with multiple independent prospective studies. Future work will aim to apply this metadata framework to a wider range of sensor data and study designs, to identify how this framework could inform future efforts to standardize metadata for DHTs.

The proposed metadata framework provides a functional method for metadata collection in a manner that is agnostic to a given study design. Additionally, the modular structure of framework has flexibility to accommodate future expansion where required. The metadata framework achieves this using three specific innovations. First, it captures the core information needed to optimize the value of the measures derived from a DHT. Second, it supports comparison of measures of the same concept of interest using different DHTs (such as APDM sensors and Apple Watch sensors in the example given above), helping us move towards a device-agnostic approach to measurement of a given concept of interest. Third, through the use of pre-specification, it provides a means to standardize and assure the quality of data collected with a DHT. Taken together, the elements of the proposed metadata framework represent an initial step toward standardization of data collection across devices and studies, paving the way greater regulatory acceptability of DHTs in clinical trials or research.

## Figures and Tables

**Table 1:** Table of metadata concepts for WATCH-PD.

Device					
Metadata Name		Pre-Specified Value?	Application-Dependent?	Metadata Item APDM	Metadata Item WATCH-PD
Device UID		No	No		
Brand		Yes	No	APDM	Apple
Model		Yes	No	Opal	iPhone and Apple Watch
Hardware version		Yes	No	Opal v.2	Apple Watch Series 5 Apple iPhone 11
Firmware version		Yes	No	20190315	Apple Watch Series 5 Apple iPhone 11
Body site		Yes	No	Each wrist	Most affected wrist (Apple Watch)
Orientation		Yes	No	Sensors are positioned with device’s charging ports oriented toward more distal body locations	Watch orientation set during initial setup, with watch ‘crown’ positioned facing the hand. Watch on most-affected side
Hub/App		Yes	No	Mobility Lab Hub	BrainBaseline version ‘WATCH-PD’
Metadata version		Yes	No	V1.0	V1.0
**Sensor and Signal**					
Sensor type		Yes	No	Accelerometer, Magnetometer, Gyroscope, Barometer	Accelerometer, Gyroscope
Recording mode		Yes	No	Active	Active
Calibration		Yes	No	Opal calibration process	N/A
Outputs	Datatype	No	No	Numeric	Numeric
	Serialization	No	No	TBD	TBD
	Units	No	No	^o^s (gyro); m/s^2^ (accel); pT (mag); mPa (bar)	^o^s (gyro); m/s^2^ (accel)
	Scaling	Yes	No	Accelerometer—100 um/s^2^ Gyroscope—10 urad/s Magnetometer—100 pT Barometer—100 mPa	-
	Filename of data file	No	No		
Data rate		Yes	No	128 Hz from all sensors	100 Hz accel and gyro in clinic 50 Hz accel in home-base passive monitoring
Timing		Yes	No	Continuous data collection from all sensors during clinic assessment	Continuous data collection during clinic assessment. 7 day home based data collection
Multiple data streams		Yes	No	Accelerometer [xyz], Magnetometer [xyz], Gyroscope [xyz], Barometer	Accelerometer [xyz], Gyroscope [xyz]
**Participant/Population**					
Subject UID		No	No		
Demographics Inclusion/exclusion criteria		Yes	Yes	Inclusion criteria from WATCH-PD protocol	Inclusion criteria from WATCH-PD protocol
Comorbidities		No	Yes	Not available	Not available
**Analysis Metadata**					
Data pre-processing		Yes	No	Not available	Not available
Endpoint analysis		Yes	Yes	WATCH-PD SAP v1.0	WATCH-PD SAP v1.0
**Experiment**					
Experiment UID		Yes	No	WATCH-PD	WATCH-PD
Protocol		Yes	Yes	WATCH-PD Protocol v3.0	WATCH-PD Protocol v3.0
Questionnaires and scales		Yes	Yes	MDS-UPDRS 2.10	In-clinic: MDS-UPDRS 2.10 In-home: Single item 7-point Likert response indicating current tremor severity
Clinical assessments/Human observation		Yes	Yes	MDS-UPDRS 3.15, 3.16, 3.17; RUE ^1^ and LUE ^2^ assessments only	In-clinic: MDS-UPDRS 3.15, 3.16, 3.17; RUE and LUE assessments only In-home: N/A—No human observation during home assessments
Clinical follow-up frequency as a comparator for digital measures		Yes	Yes	Baseline, 1 month, 3 month, 6 month, 9 month, 12 month	Baseline, 1 month, 3 month, 6 month, 9 month, 12 month
Clinical correlations to digital measures		Yes	Yes	Sensor collection simultaneous with clinical assessments described above	In clinic, sensor collection simultaneous with clinical assessments described above
Active test details		Yes	Yes	Sensor collection simultaneous with clinical assessments as per protocol.	In clinic, sensor collection simultaneous with clinical assessment as per protocol.
Passive monitoring		Yes	Yes	N/A	At Home, 7 days passive monitoring.
**Contextual Data**					
Environment		Yes	Yes	Clinic	Clinic, home
Environmental context (carer, temperature, indoor/outdoor, …)		Not available	Yes	Not available	Not available

^1^ RUE: right upper extremity; ^2^ LUE: left upper extremity; N/A: not applicable.

## Data Availability

Data presented in this paper are from an ongoing study and serve the sole purpose to support a conceptual framework with examples. No data of the currently ongoing study can be shared until completion and dissemination of results and approval by study sponsor.
